# Early childhood development status and associated factors among preschool children attending routine well clinics in Temeke Municipal, Dar es Salaam-Tanzania: a cross-sectional study

**DOI:** 10.11604/pamj.2024.49.137.45990

**Published:** 2024-12-31

**Authors:** Mufida Ali Mshimba, Florence Salvatory Kalabamu, Maulid Fataki, Leonard Malasa, Felician Rutachunzibwa

**Affiliations:** 1 Department of Pediatrics and Child Health, Kairuki University, Dar es Salaam, Tanzania

**Keywords:** Early childhood development, Temeke, Dar es Salaam

## Abstract

**Introduction:**

Early Childhood Development (ECD) is a social and public health concern especially in low- and middle-income countries whereby around 43% of children living in these countries are at risk of developmental delays. This may negatively affect their potential including reduced productivity in adulthood. Data from the 2022 Tanzania Demographic and Health Survey has shown that around 47.4% of children aged 24-59 months scored low in their early childhood scores. However, factors associated with low suboptimal ECD are not well understood. This study aimed to determine the magnitude and characteristics associated with low ECD scores among children aged 24-59 months attending RCH clinics in Temeke Municipal, Dar-es-salaam region.

**Methods:**

the study was a facility-based cross-section study design involving Children aged 24 - 59 months attending RCH clinics in Temeke District, Dar-es-salaam, Tanzania. An interviewer-guided questionnaire was used to collect basic demographic information while ECD scores among participants were determined using a standardized ECD-I2030 tool. Data analysis was conducted using Statistical Package for Social Sciences (SPSS). The magnitude of children not on track was expressed in frequency and percentages. Factors associated with poor ECD were determined by using binary logistic regression analysis. The alpha level of 0.05 or less was considered statistically significant.

**Results:**

a total of 422 children were enrolled in the study. Among participants, only 144(34.1%) were on track based on their childhood development scores while 278 (65.9%) were off track. Young age (AOR=0.149 (0.354-0.63); p-value=0.001), nutritional status (AOR=7.729(2.234-26.735); p value=0.010) and parents' employment status (AOR=3.730(1.937-7.184); p-value=0.001) were independently associated ECD scores.

**Conclusion:**

most children enrolled in this study were off-track in their ECD scores which may limit the realization of their full potential. Young age, malnutrition and unemployed parents were significant factors associated with EDC delays. Therefore, targeted interventions aimed at ensuring food security and nutrition, economic empowerment of families through formal employment, and early positive parenting practices may improve ECDs among this age group.

## Introduction

Early Childhood Development (ECD) are changes that take place from conception up to 8 years of age, with more emphasis on the first 1,000 days of life [1]. This is the period of rapid brain development, with maximum capacity to form multiple neuronal connections in response to internal and external experiences [1,2]. It is estimated that during this period, more than 1 million new neuronal connections are made per second, making it a crucial period of both great risk and opportunity for survival and well-being from in-utero to adulthood [2]. These ECD changes take place in different domains such as physical, cognitive, psychosocial and language development [2,3]. Studies have shown that good nutrition, healthcare, protection from physical and emotional harm, responsive caregiving, early learning, and a stimulating environment positively influence ECD [2,4]. These early changes provide a foundation stone for well-being and productivity throughout the entire life course, making ECD the foundation of community and global prosperity. Notably, it is estimated that investment in ECD initiatives may increase the individual ability to earn income by 25% during adulthood, with a return on invested capital of 13% [3,5].

Investing in early childhood development leads to multiple yields for respective individuals and the entire societies. To ensure this, the Sustainable Development Goal (SDG) number 4.2.1 underpins the importance of ECD by ensuring that children aged 24-59 months are developmentally on track on health, learning, and psychosocial wellbeing [6]. Despite the crucial influence of ECD in life, it is estimated that 43% of children under 5 years of age, translating into 250 million under-fives living in the Lower-and-Middle Income Countries (LMIC) are at the risk of not attaining their full potential [7]. Similarly, results from the year 2022 Tanzania Demographic, Health and Malaria Indicator Survey (TDHS-MIS) estimated that only 47% of children between 24 and 59 months were on track in both health, learning, a social well-being [8]. Suboptimal ECD in LMIC is directly linked to a higher presence of early childhood adverse experiences such as extreme poverty, crisis (wars and domestic violence), discrimination, and disability [7,9,10].

Poor ECD in resource-constrained countries increases the risk of not achieving their potential, which is detrimental to them, and to the country's progress towards the 2030 sustainable development goals as the result of poor growth, mental health, poor school performance, inadequacy of social skills, and poor economic productivity potential [11]. Even though the major barricades for ECD are known, the higher percentage of children in Tanzania are off track in ECD which necessitates designing and accelerating targeted interventions aimed at promoting ECD. However, this can be well implemented if the local contextual factors associated with poor ECD are well-known and mitigated. Therefore, in this study, we aimed to assess the ECD status and associated factors among children aged 24 to 59 months in Temeke, Dar es Salaam.

## Methods

**Study design:** this study was a cross-sectional hospital-based study conducted in Temeke Municipal, which is among the five municipals in Dar es Salaam metropolitan city.

**Study setting and population:** according to the national 2022 census, Temeke is estimated to have a total population of 1.3 million with an estimated annual growth rate at 4.6% [12]. Using a multistage stratified sampling technique, four health facilities (Buza, Arafa, Round Table, and Pasada) were selected from a list of health facilities in Temeke municipal. Children aged 24 to 59 months were recruited from routine well clinics from the respective health facilities.

**Inclusion and exclusion criteria:** the inclusion criteria were children aged 24-59 months with their primary care givers, being a resident of Temeke municipality for at least 6 months, and informed consent from primary care givers. Children with congenital malformations, severe acute illnesses, and other disabilities were excluded from the study.

**Sample size:** a total sample size of 422 was calculated using the Cochrane formula for determining the proportion from infinite proportion [13]. A number of participants to be enrolled from each facility was obtained using the ratio of estimated aggregate number of children attending the selected health facilities estimated from the previous year's facility records. Therefore, 153 participants were enrolled from Buza, 99 from Arafa, 92 from Round Table, and 77 from Pasada.

**Study variables and data collection:** social demographic data were collected using an interviewer-guided pre-structured questionnaire. Variables collected include age of the child, sex, nutritional status, gestation age at birth, education, and duration of exclusive breastfeeding. Additionally, caregivers' age, level of education, occupation, and residence were collected. ECD scores were calculated using the Early Childhood Development Score Index (ECDI-2030) developed and validated by UNICEF [14], designed specifically for countries to monitor progress of SDG target 4.2. Moreover, this tool considers the context of lower and moderate-income countries for children between 24-59 months, focusing on four major domains: health, education, psychology, and social well-being.

**ECD scores assessment:** the questionnaire for social demographic information, and the ECD scores chart with 20 items were administered among primary caregivers with their children in a comfortable and private room at the respective health facilities. Caregivers were guided to give details on how their children behave daily, routine abilities and knowledge. A child was considered to be on track on ECD if he/she scored at least 7 items (children between 24-29 months); 9 items for 30-35months; 11 items for 36-41 months, 13 for 42-47 months, and 15 items for 48-59months as per ECDI-2030 manual [14].

**Assessment of nutritional status:** assessment of the nutritional status of children was performed using anthropometric measurements and interpreted using the WHO weight for height z-score charts [15,16]. Weight for each participant was measured using SECA weighting scale (SECA diagnostics, Hamburg, Germany). The weight was measured to the neared 0.1 kilogram while the child standing straight on the scale as per standard UNICEF/WHO guideline [17] while height was measured using a wooden stadiometer while the child standing straight as per standard WHO guideline [17]. The child was categorized as wasted if the score corresponded to less or equal to -2 standard deviation while equal or more than +2 were considered to be overweight.

**Data analysis:** data analysis was done using Statistical Software for Social Sciences (SPSS) version 20 (IBM SPSS Inc, Chicago, USA). Continuous variables were summarized using means with standard deviations or medians and interquartile range while the magnitude of children on track was calculated as frequency and respective percentages. Binary logistic regression analysis was used to assess socio-demographic factors associated with poor EDC status. The binary logistic model was conducted in two steps: firstly, univariate analysis was conducted by assessing the relationship between one independent variable and the dependent variable (ECD status) at a time. Crude Odd Rations (COR) and respective 95% confidence intervals and respective p values were calculated. Secondly, independent variables with a significant level at less or equal to 0.2 in the first step were included in the multivariate model using the “enter” method. Adjusted Odd Rations (AOR) with respective 95% confidence intervals and p-values were computed. Variables with alpha levels of less or equal to 0.05 in the multivariate model were considered to be independently associated with poor ECD status.

**Ethical considerations:** this study was conducted as per international guidelines for conducting health research using human subject as stipulated in the declaration of Helsinki and as per Tanzania guidelines of conducting health research. The study was reviewed and approved by the institutional review committee of the Kairuki University with reference number HKMU/IREC/27.10/395. Furthermore, permission to conduct the study was sought from the Temeke Municipal Health Administration Office and the respective facility administration. To ensure free participation the informed consent was sought from the parents/guardians of the children before enrollment. Additionally, to ensure confidentiality interviews and ECD assessment were conducted in a private room located at the facility with only the parents/guardians and the child present in the room. A child who was found to be lagging behind in ECD scores was referred to the existing care system for further assessment and management after thorough parental counselling. No personal identifiers were used in data collection and analysis code numbers were used instead of names, and data analysis was conducted anonymously.

## Results

A total of 422 participants were enrolled in the study. Among these 245(58.1%) males while 177(41.9%) were females. The average age for participants was 36.8 ± 9 months. Additionally, 77(18.2%) were attending school at the time of data collection. At the time of data collection, the majority of participants (73%) were under the daily care of their mothers. Other demographic characteristics of participants are shown in Table 1. Among the mothers of study participants 260(61.6%) had primary level education. Moreover 18(4.3%) of the mothers had higher education training compared to 36(8.5%) among fathers of the children involved in the study. Furthermore 278(65.9%) of mothers had no specific means of earning compared to 165 (39.1%) of fathers (Table 2).

**Table 1:** baseline demographic characteristic of study participants

Variable		Frequency	Percent
**Gender**	Male	245	58.1
	Female	177	41.9
**Age groups (Months)**	24-29	107	25.4
	30-35	113	26.8
	36-41	84	19.9
	42-47	42	10.0
	48-59	76	18.0
**Gestation age at birth**	Pre-term	7	1.7
	Term	415	98.3
**Exclusive breastfeeding**	Yes	232	55
	No	190	45
**Birth weight**	Below 2.5	31	7.3
	2.5 and above	391	92.7
**Attending school**	Yes	77	18.2
	No	345	81.8
**Childcare options (During the daytime)**	School	72	17.1
	Housemaid	8	1.9
	Mother	308	73.0
	Father	4	0.9
	Others	30	7.1
**Nutritional status**	Normal	324	77
	Moderate malnutrition	49	12
	Severe malnutrition	48	11
	**Total**	**422**	**100.0**

**Table 2:** social demographics characteristics of parents

Variable	Frequency	Percent	Frequency	Percent
	Mothers		Fathers	
**Age groups**				
Below 20	2	.5	0	0
20-24	85	20.1	8	1.9
25-29	164	38.9	91	21.6
30-34	98	23.2	122	28.9
35-39	38	9.0	81	19.2
40-44	23	5.5	62	14.7
45 and above	12	2.8	58	13.7
**Educational Level**				
Primary	260	61.6	218	51.7
Secondary	144	34.1	168	39.8
Higher education	18	4.3	36	8.5
**Employment Status**				
Non-employed	278	65.9	165	39.1
Employed	79	18.7	149	35.3
Self employed	65	15.4	108	25.6
**Total**	**422**	**100.0**	**422**	**100.0**

**Early childhood development status:** among all study participants 278(65.9%) were not on track on ECD milestones. Among children not on track 172(61.9%) were males. On the binary logistic regression model, children from young age groups (less than 36 months) were at lower risk of poor EDC compared to the older age groups. Similarly severe malnutrition was an independent risk of poor ECD status; AOR=7.72 (2.23-26.73), P<0.001'> P<0.001] (Table 3).

**Table 3:** children's factors associated with poor ECD status among participants (N=422)

Variables		ECD Status		COR (95%CI)	p-value	COR (95% CI)	p-value
		On Track	Not on Track				
		n (%)	n (%)				
**Age groups (in months)**	24-29	58 (54.2)	45(45.8)	0.17(0.08 -0.35)	<0.001	0.01 (0.04-0.23)	<0.001
	30-35	49 (43.4)	64(56.6)	0.27(0.13-0.54)	<0.001	0.14 (0.06-0.35)	<0.001
	36-41	15(17.9)	69(82.1)	0.94(0.41-2.15)	0.9	0.61 (0.24-1.53)	0.28
	42-47	9(21.4)	33(78.6)	0.75(0.29-1.95)	0.76	0.63 (0.22-1.78)	0.39
	48-59	13(17.1)	63(82.9)	Ref.	-	-	-
**Sex**	Male	73(29.8)	172(70.2)	1.57(1.05-2.37)	0.02	1.43 (0.90-2.27)	0.22
	Female	71 (40.1)	106(59.9)	Ref	-	-	-
**Gestational age at birth**	Preterm	4 (57.1)	3(42.9)	0.83(0.08-1.73)	0.21	0.47(0.65-3.43)	0.45
	Term	140 (33.7)	275(66.3)	Ref	-	-	-
**Exclusive breastfeeding**	Yes	81(34.9)	151(65.1)	1.08(0.72-1.62)	0.7	0.91(0.57-1.45)	0.71
	No	63(33.2)	127(66.8)	Ref	-	-	-
**Birth weight**	< 2.5 kg	15 (48.4)	16(51.6)	1.90 (0.91-3.97)	0.08	0.86(0.34-2.21)	0.76
	≥ 2.5 kg	129(33.0)	262(67.0)	Ref.	-	-	-
**Nutritional status**	Severe malnutrition	3(6.3)	45(93.8)	10.18(3.09-33.45)	<0.001	7.72(2.23-26.73)	0.01
	Moderate malnutrition	10(20)	40(80)	2.17(1.31-5.62)	0	2.04(0.93-4.48)	0.07
	Normal nutrition	131(40. 4)	193(59.6)	Ref.	-	-	-
**School attendance**	yes	33(42.9)	44(57.1)	1.58(0.95-2.61)	0.07	0.47(0.77-2.90)	0.41
	No	111(32.2)	234(67.8)	Ref.	-	-	-
**Childcare during the day**	School	31(43.1)	41(56.9)	0.76(0.31-1.84)	0.55	0.78(0.10-6.22)	0.82
	Housemaid	2(25.0)	6(75.0)	1.73(0.29-1.13)	0.54	3.84(0.54-27.18)	0.17
	Mother	99(32.1)	209(67.9)	1.22(0.56-2.66)	0.61	1.54(0.62-3.81)	0.34
	Father	1(25)	3(75.0)	1.73 (0.161.80)	0.65	1.36(0.99-18.89)	0.81
	Others	11(36.7)	19(63.3)	Ref.	-	-	-

On assessing parental factors associated with poor EDC status, children whose mothers were employed had higher odds of having poor ECD status compared to children from mothers who were self-employed; AOR=5.67 (2.40-13.41), p<0.001. Moreover, compared to children from mothers who were self-employed, children from unemployed mothers had higher odds of having poor ECD status; 2.29(1.16-4.50), p<0.001. Similarly, children from unemployed fathers had higher odds of poor ECD status compared to children from self-employed mothers; 3.73(1.93-7.18), p<0.001 (Table 4).

**Table 4:** parents' factors associated with poor ECD among study participants (N=442)

Variables		ECD Status		COR (95% CI)		p-value	AOR (95% CI)	p-value
Age groups Mothers in years		On Track	Not on Track					
		n (%)	n (%)					
	Below 20	0(0.0)	2(100)	Reference				
	20 – 24	28(32.9)	57(67)	0.7(0.17-2.70)		0.58	0.50(0.08-2.89)	0.44
	25 – 29	55(33.5)	109(66.5)	0.66 (1.17-2.53)		0.54	0.35(0.06-1.89)	0.22
	30 -34	39(39.8)	59(60.2)	0.50(0.12-1.98)		0.32	0.27(0.05-1.36)	0.11
	35 -39	11(28.9)	27(71.7)	0.81(0.18-3.60)		0.79	0.49(0.09-2.58)	0.4
	40-45	8(34.8)	15(65.2)	0.62(0.13-2.98)		0.55	0.53(0.09-2.97)	0.47
	≥ 45	3(25.0)	9(75.0)	0.39(0.09-1.68)		0.2	0.31(0.05-1.73)	0.18
Age groups Fathers in years	Below 20	1(12.5)	7(87.5)	Reference				
	20 - 24	29(31.9)	62(68.1)	1.40(0.70-2.79)		0.33	1.78(0.60-5.29)	0.29
	25 – 29	48(39.3)	74(60.7)	1.01(0.53-1.92)		0.97	1.59(0.58-4.33)	0.35
	30 -34	24(29.6)	57(70.4)	1.561(0.76-3.17)		0.21	2.91(1.11-7.62)	0.03
	35 -39	23(39.7)	35(60.3)	1.02(0.55-1.88)		0.94	1.67(0.63-4.44)	0.29
	40-44	23(39.7)	35(60.3)	1.02(0.55-1.88)		0.94	1.67(0.63—4.44)	0.29
	≥ 45	9(42.7)	12(57.1)	1.66(0.57-4.85)		0.35	2.37(0.67-8.31)	0.17
Level of education of mothers	Primary	84(32.3)	176(67.7)	0.89(0.27-2.33)		0.69	0.32(0.07-1.32)	0.11
	Secondary	55(38.2)	89 (61.8)	0.62(0.21-1.84)		0.39	0.33(0.08-1.34)	0.12
	Higher education	5(27.8)	13(72.2)	Reference				
Level of education of fathers	Primary	57(26.1)	161(73.9)	2.52(1.22-5.19)		0.01	2.71(0.95-7.75)	0.06
	Secondary	70(41.7)	98 (58.3)	1.25(0.60-2.58)		0.54	1.88(0.70-5.02)	0.2
	Higher education	17(47.2)	19 (52.8)	Reference				
Employment status (Mothers)	Non-employed	85(30.6)	193(69.4)	4.3 (2.5-7.9)		<0.001	2.29(1.16-4.50)	<0.001
	Employed	16(20.3)	63(79.7)	7.69(3.62-16.32)		<0.001	5.67(2.40-13.41)	<0.001
	Self employed	43(66.2)	22(33.8)	Reference				
Employment Status (Fathers)	Non-employed	57(26.1)	161(73.9)	5.07(2.91-8.84)		<0.001	3.73(1.93-7.18)	<0.001
	Employed	70(41.7)	98(58.3)	1.49(0.9-2.46)		0.11	1.14(0.63-2.07)	0.64
	Self-employed	17(47.2)	19(52.8)	Reference				

## Discussion

In this study, we aimed to determine the magnitude of poor ECD among preschool children attending routine well-being clinics in Temeke municipal in Dar es Salaam. We found that 65.9% of children in this study were lagging behind in ECD status. Age above 36 months, malnutrition, and lack of reliable source of income among parents were independently associated with poor ECD status.

The high magnitude of children with poor ECD status in this study is consistent with the most recent findings from the year 2022 Tanzania Demographic, Health and Malaria Indicator Survey which showed that 53% of children of the same age group who were involved in a national survey were not on track in both health, learning, a social well-being [8]. Moreover, this finding reflects the 2016 Lancet Series projections indicating that almost 43% of children in low-and middle-income countries were at higher risk of suboptimal development based on stunting and poverty [7].

We found that age was highly associated with poor ECD status whereby children younger than 36 months were less affected. However, the risk could have started early and unfolded as they grew up. This emphasizes the importance of starting ECD interventions as early as possible to ensure the best possible life starts, preferably from pregnancy to 3 years of age as this is the most sensitive period for brain neuronal connections [1]. We also found that 70.6% of children above 36 months of age were not attending pre-primary (nursery) care. This is contrary to the nurturing care framework by UNICEF which recommends accessibility of quality pre-primary education services and community-based day care programmes for children from 3 to 6 years of age [18]. Therefore, this delayed opportunity for learning could have also contributed lagging behind in ECD scores among this age group.

Our study findings are consistent with findings from similar studies conducted elsewhere where malnutrition was highly associated with poor ECD index [19-21]. This further supports the use of stunting as the proxy measure for potential risk for poor ECD status employed by WHO and the Lancet series on ECD [4,7,22] . Even though we only performed anthropometric measurements to assess macronutrient status, macronutrient deficiency is usually accompanied by micronutrient deficiency [23,24]. Recent studies conducted in a similar setting showed a significant level of iron deficiency anemia which is also a potential risk for poor motor, cognitive, and socio-emotional development [25,26].

This study is in line with the UNICEF nurturing care for early childhood development framework which emphasizes proper nutrition for mothers and children [18]. This includes supplements with micronutrients and minerals such as iron during pregnancy, followed by exclusive breastfeeding for at least 6 months and complementary feeding thereafter with food rich in diverse nutrients for optimal growth [18]. Henceforth, our findings underline the importance of appropriate nutrition and ensuring food security for children and families.

Similar to other studies [27], we found that low earning ability and unemployment status among parents increased the odds of poor ECD among their children. Employment is directly related to the earning ability of individuals and families, with increased capacity to take care of the most vulnerable members of families, including children. This includes the provision of appropriate food, healthcare and physical protection which are essential components of nurturing care framework.

The use of the standardized and validated tool (ECDI2030) strengthens our study results making them easily compared with other similar studies using the same tool. However, essential information for ECD scoring were provided by caregivers, which is a potential risk for recall bias and misinterpretation. To minimize this limitation, we used well-trained research assistants, and the data collection tool was translated into local language (Swahili) for easy understanding. Secondly, the tool guide provides aggregate scores which limits comparisons of ECD in specific domains which could limit information for designing specific interventions targeting specific domains.

## Conclusion

In conclusion, the magnitude of poor ECD developmental status was high with malnutrition, age, and employment status independently associated with poor ECD status. This may limit children from achieving their full potential, which subsequently limits the wellbeing of individuals, communities, and the country. Therefore, to foster EDC, and achieve SDG on ECD, there is a need to design and implement ECD interventions early in life using multisectoral approaches including improving nutrition for pregnant mothers and children, positive parenting education, and economic empowerment of families. In addition, to inform on designing and implementing targeted interventions, further studies should be conducted to determine the most affected ECD domains and appropriate interventions.

### What is known about this topic

- Early childhood experiences influence physical, mental, and social development of children;
- Children in lower- and middle-income countries are at higher risk of suboptimal ECD with subsequent risk of not attaining their full potential due to high level of poverty, malnutrition, and family-social conflicts;
- Nurturing care comprising of good nutrition, healthcare, safe environment, responsive caregiving, and early opportunity for learning are crucial for optimal ECD.

### What this study adds

- This study highlights high magnitude of children who are lagging behind in ECD in limited resources setting in Tanzania;
- Children's age, nutrition status, and parental employment play a great role in ECD in limited resources like in Temeke, Tanzania.
